# Clinical characteristics of COVID-19 complicated with pleural effusion

**DOI:** 10.1186/s12879-021-05856-8

**Published:** 2021-02-15

**Authors:** Na Zhan, Yingyun Guo, Shan Tian, Binglu Huang, Xiaoli Tian, Jinjing Zou, Qiutang Xiong, Dongling Tang, Liang Zhang, Weiguo Dong

**Affiliations:** 1 Department of Pathology, Renmin Hospital of Wuhan University, Wuhan, China; 2 Department of Gastroenterology, Renmin Hospital of Wuhan University, 99 Zhangzhidong Road, Wuhan, 430060 Hubei Province China; 3 Department of Respiratory Medicine, Renmin Hospital of Wuhan University, Wuhan, China; 4 Department of Clinical Laboratory, Renmin Hospital of Wuhan University, Wuhan, China; 5 Department of Radiology, Renmin Hospital of Wuhan University, Wuhan, China

**Keywords:** COVID-19, Pleural effusion, Prognosis, Predictive model, Risk factor

## Abstract

**Background:**

Epidemiological and clinical features of patients with corona virus disease 2019 (COVID-19) were well delineated. However, no researches described the patients complicated with pleural effusion (PE). In the present study, we aimed to clinically characterize the COVID-19 patients complicated with PE and to create a predictive model on the basis of PE and other clinical features to identify COVID-19 patients who may progress to critical condition.

**Methods:**

This retrospective study examined 476 COVID-19 inpatients, involving 153 patients with PE and 323 without PE. The data on patients’ past history, clinical features, physical checkup findings, laboratory results and chest computed tomography (CT) findings were collected and analyzed. LASSO regression analysis was employed to identify risk factors associated with the severity of COVID-19.

**Results:**

Laboratory findings showed that patients with PE had higher levels of white blood cells, neutrophils, lactic dehydrogenase, C-reactive protein and D-dimer, and lower levels of lymphocytes, platelets, hemoglobin, partial pressure of oxygen and oxygen saturation. Meanwhile, patients with PE had higher incidence of severe or critical illness and mortality rate, and longer hospital stay time compared to their counterparts without pleural effusion. Moreover, LASSO regression analysis exhibited that pleural effusion, lactic dehydrogenase (LDH), D-dimer and total bilirubin (TBIL) might be risk factors for critical COVID-19.

**Conclusions:**

Pleural effusion could serve as an indicator for severe inflammation and poor clinical outcomes, and might be a complementary risk factor for critical type of COVID-19.

## Background

An epidemic of Coronavirus Disease 2019(COVID-19) struck Wuhan, China and rapidly spread to the entire country and around the globe [1–3]. Till August 31, 2020, A cumulative total of nearly 25 million cases and 800,000 deaths globally were reported since the start of the outbreak according to the World Health Organization [4] and the National Health Commission of China reported a total of 85,058 confirmed COVID-19 cases, 4634 deaths, and 80,208 cured cases in China [5, 6]. COVID-19 was caused by the severe adult respiratory syndrome coronavirus 2 (SARS-CoV-2). The diagnosis of COVID-19 was established on the basis of contact history, clinical features, imaging findings and results of RT-PCR tests [7]. Given the wide clinical spectrum of COVID-19, understanding the factors that can predict disease severity were essential since this would help frontline clinical staff to stratify patients with increased confidence [8]. Pleural effusion (PE), lung cavitation, lymphadenopathy and calcification were rarely seen in COVID-19 patients [9–11]. Previous studies demonstrated that PE exerted a significant influence on the final outcome of patients suffering from acute lung injury or acute respiratory distress syndrome [12]. Recent study found that severe/critical patients showed more lymph node enlargement, pericardial effusion, and pleural effusion, which suggesting these extrapulmonary lesions may indicate the occurrence of severe inflammation, However, the sample size of that research was relatively small [13]. Additionally, The possibility of PE prediction for progression to critical condition of COVID-19 patients was not yet analyzed.

In the present study, we preliminarily characterized the imaging findings of 476 COVID-19 patients. Then, we compared COVID-19 patients with and without PE in terms of their clinical futures and outcomes. Finally, a predictive model based on PE and other clinical features was created to identify COVID-19 patients who may progress to critical condition.

## Methods

### Patient selection

**Fig. 1:**
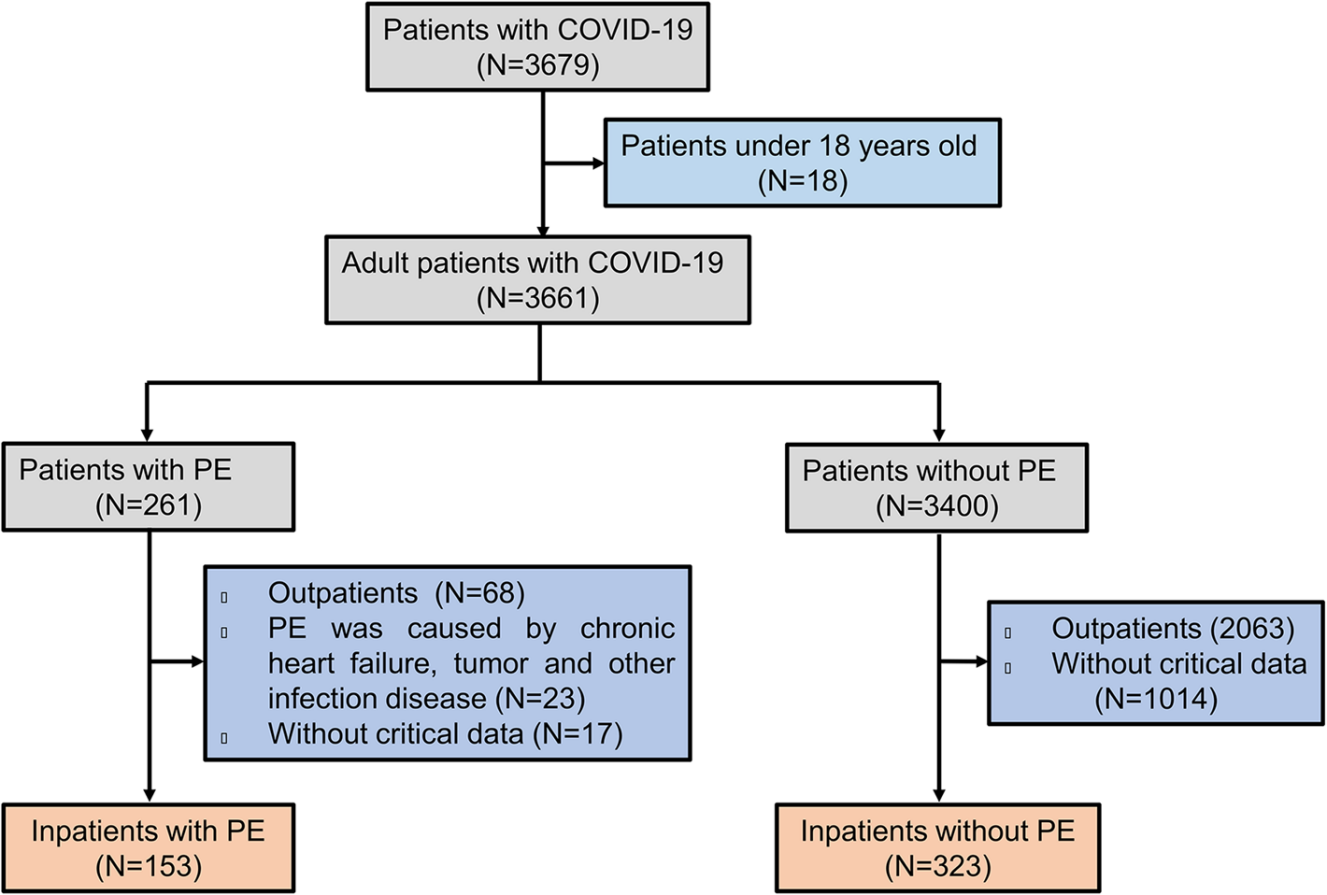
Flowchart of COVID-19 patient inclusion

This project was a retrospective single-center study, which included 476 COVID-19 patients hospitalized in Renmin Hospital of Wuhan University (Wuhan, Hubei province, China) from January 20, 2020 to March 23, 2020. Patients were excluded from the study if they met any of the following criteria: (1) age < 18 years; (2) relevant data were not available; (3) PE was caused by chronic heart failure, malignant tumors, tuberculosis and other infection diseases by clinical history, imaging examination or thoracentesis. Severe and critical cases were defined according to the guidelines of COVID-19 diagnosis and treatment plan (trial version 8) developed by the National Health Commission of China (http://www.nhc.gov.cn/). The patients were categorized as follows: (1) general type: patients have fever, respiratory symptoms, and imaging findings of pneumonia. (2) severe type: patients have one of the following: (a) respiratory distress, respiratory rate ≥ 30 beats / min; (b) resting oxygen saturation ≤ 93%; (c) arterial blood oxygen partial pressure / oxygen concentration ≤ 300 mmHg (1 mmHg = 0.133 kPa). (3) critical type: cases have one of the following features: (a) respiratory failure and need for mechanical ventilation; (b) shock; (c) organ failure requiring intensive care. All 476 patients with COVID-19 were divided into two groups in terms of CT findings. Group 1 included 153 patients with PE while Group 2 had 323 patients without PE. Flowchart for patient selection is shown in Fig. 1. The study was conducted in accordance with the principles of the Declaration of Helsinki, and was approved by the ethics committee of Renmin Hospital of Wuhan University (NO. WDRY2020-K128). Written informed consent was obtained from each participating patient.

### Image acquisition and analysis

Chest CT scan was performed in all 476 patients with COVID-19 by employing an Optima CT680 scanner (GE Medical Systems, Milwaukee, WI), which set at 210 mA and 120 kV with a minimum slice thickness of 1 mm. All images were analyzed in a consistent manner by two experienced chest radiologists. Image analysis was based on the lesion features of each patient including: (a) lesion distribution, (b) number of involved lobes, (c) lobe of lesion distribution, (d) lesion patters(e.g., ground glass opacities, pulmonary consolidation, linear opacities) and (e) other findings (e.g., adjacent pleural thickening, pleural effusion, pericardial effusion, thoracic lymphadenopathy, pulmonary emphysema) [14]. The alterations caused by underlying lung diseases such as tuberculosis and lung cancer were excluded in this study.

### Data collection

The demographic data, clinical features (including medical history, comorbidities, signs and symptoms), laboratory findings and chest CT results were obtained through electronic admission records. Information on date of symptom onset, initial clinic visit, hospital admission, result of SARS-CoV-2 RNA detection, and the type of COVID-19 was also taken. The onset date was defined as the date when symptoms were noticed. Data were reviewed by a trained team of experienced physicians and independently analyzed by three researchers.

### Diagnostic test for COVID-19

Nasopharyngeal and oropharyngeal swab specimens were collected and tested by fluorescence RT-PCR assay by using a SARS-CoV-2 RNA kit (Shanghai Geneodx Biotech Co. Ltd.), approved by National Medical Products Administration (NMPA) and recommended by Chinese Centers for Disease Control and Prevention (CDC) [15].

### Statistical analysis

Continuous variables were presented as means and standard deviations, or medians and interquartile range (IQR) values. Categorical variables were expressed as counts and percentages. When the data were normally distributed, independent t-tests were employed to compare the mean of continuous variables. Otherwise, the Mann-Whitney test is adopted. The χ2 test was applied to compare the proportion of categorical variables. LASSO regression analysis was conducted to select independent risk factors for critical COVID-19. ROC curve was plotted and area under curve (AUC) was measured to evaluate the predictive power of the model. Statistical analysis was performed by using the SPSS software package version 13.0. A *P* value < 0.05 was considered significant.

## Results

### Imaging findings of COVID-19 patients with PE

**Fig. 2:**
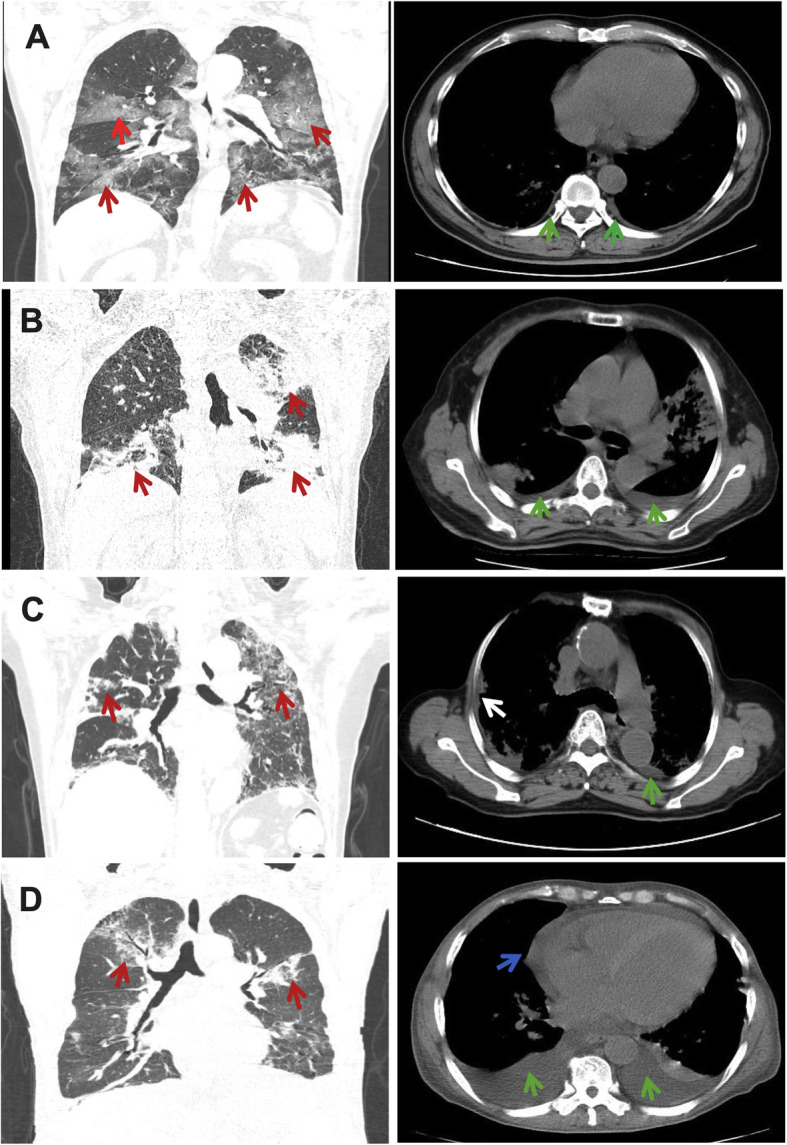
Imaging findings of COVID-19 patients with pleural effusion: **a**: Multifocal ground-glass opacities (red arrow on the coronal image) and pleural effusion (green arrow on the axial image). **b**: Multiple patchy consolidation in the upper left lobe (red arrow on the coronal image) and the lower two lobes with bilateral pleural effusion (green arrow on the axial image). **c**: Bilateral ground-glass opacities (red arrow on the coronal image) and pleural effusion (green arrow on the axial image) with pleural thickening (white arrow on the axial image). **d**: Bilateral ground-glass opacities (red arrow on the coronal image) with pleural (green arrow on the axial image) and pericardial effusion (blue arrow on the axial image)

Baseline chest CT showed that 100 of 153 patients (65.36%) developed bilateral PE. Fifty of 153 patients (32.68%) exhibited only ground glass opacities (Fig. 2a). Five patients (3.27%) showed only pulmonary consolidation (Fig. 2b) and 7 (4.58%) presented with only linear opacities. Forty patients (26.14%) displayed ground glass opacities with pulmonary consolidation. Thirty-one patients (20.26%) had ground glass opacities with liner opacities. Only 6 patients (3.92%) showed pulmonary consolidation with liner opacities. Fourteen patients (9.15%) were found to have all three lesion patterns. As for other findings, 31 of 153 patients (20.26%) were complicated with pleural thickening (Fig. 2c); 12 (7.84%) with pericardial effusion (Fig. 2d), and 8 (5.23%) suffered from pulmonary emphysema. Lymphadenopathy was uncommon in this series (Table 1).

**Table 1:** Features of chest CT scan of COVID-19 patients with Pleural effusion

	Patients(***n*** = 153)
Patterns of the lesions	
Ground glass opacities	50 (32.68%)
Consolidation	5 (3.27%)
Linear opacities	7 (4.58%)
Ground glass opacities with consolidation	40 (26.14%)
Ground glass opacities with liner opacities	31 (20.26%)
Consolidation with liner opacities	6 (3.92%)
Ground glass opacities with consolidation and liner opacities	14 (9.15%)
Pleural effusion	
Left	29 (18.95%)
Right	24 (15.69%)
Bilateral	100 (65.36%)
Other findings	
Pleura thickening	31 (20.26%)
Pericardial effusion	12 (7.84%)
Pulmonary emphysema	8 (5.23%)
Lymphadenopathy	3 (1.96%)

### Comparison of demographics and clinical indicators between COVID-19 patients with and without PE

**Fig. 3:**
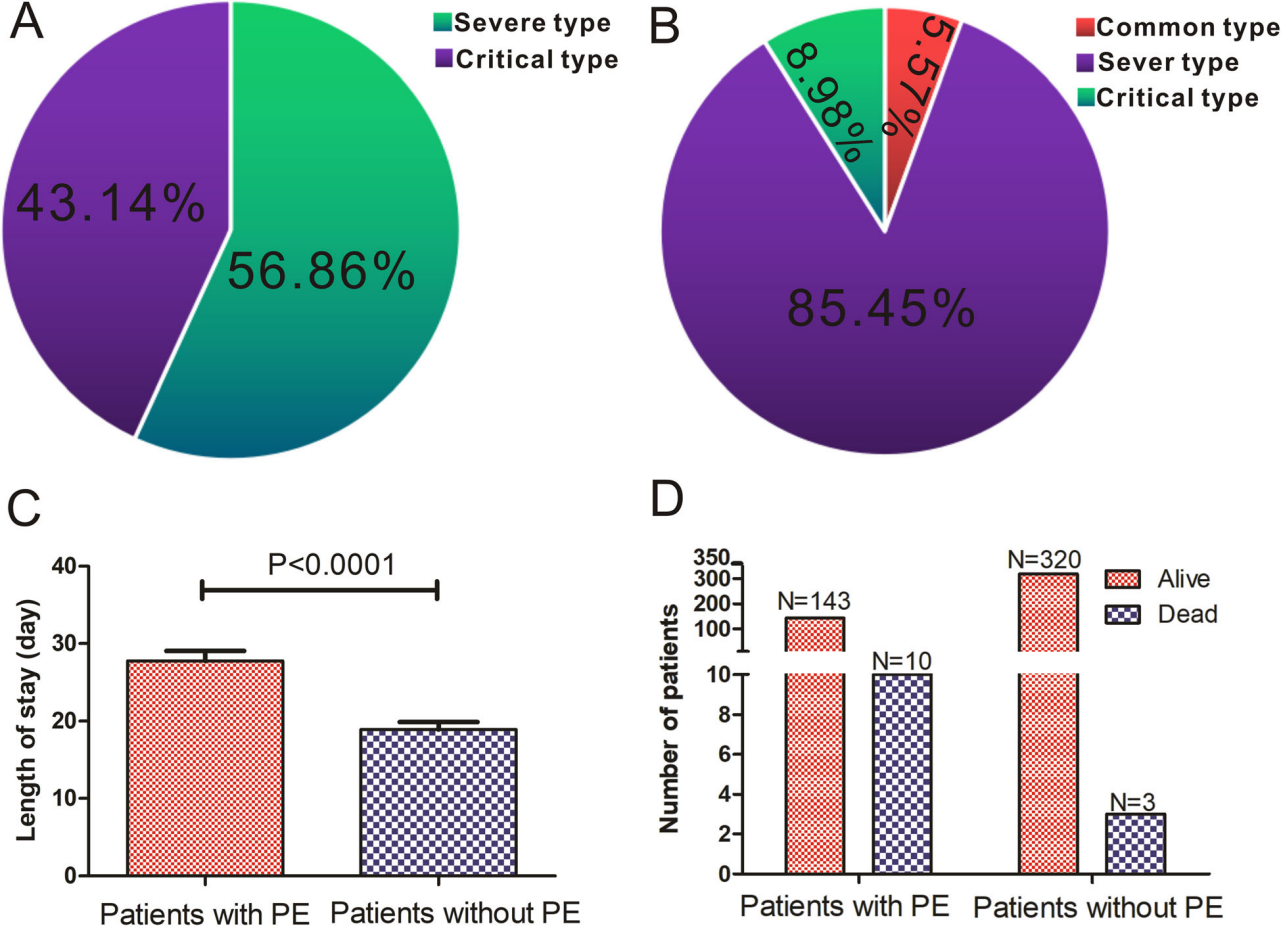
Associations between the presence of PE and clinical outcomes in patients with COVID-19. Disease conditions were different between patients with PE (**a**) and patients without PE (**b**). Length of hospital stay of patients with PE was longer than that of patients without PE (**c**). The mortality rate was significantly higher in patients with PE than in patients without PE(**d**)

All 476 patients, including 153 patients with PE and 323 without PE, were included in this study (Table 2). There existed no significant difference in most underlying diseases between PE group and none-PE group, apart from chronic disease (*P* = 0.020) and other diseases (*P* < 0.001). Meanwhile, PE group had a higher incidence of fever (*P* = 0.012), cough (*P* < 0.0001), breath shortness (*P* = 0.014) and slower heart rate (*P* < 0.0001). According to their laboratory findings, patients with PE had higher levels of white blood cells (*P* = 0.026), neutrophils (*P* = 0.012), lactic dehydrogenase (LDH, *P* = 0.001), C-reactive protein (CRP, *P* < 0.001) and D-dimer (*P* < 0.0001), and lower levels of lymphocytes (*P* = 0.043), platelets (*P* = 0.001), hemoglobin (*P* = 0.022), partial pressure of oxygen (PO_2_, *P* = 0.001) and oxygen saturation (SpO_2_, *P* = 0.001). Moreover, patients with PE had higher incidence of severe or critical COVID-19 (*P* < 0.001) and longer hospital stay time (*P* < 0.0001). By the end of March 23, 10 patients in PE group died, while only 3 patients deceased in no PE group, suggesting that the mortality rate was statistically (*P* = 0.001) higher in patients with PE than in those without PE (Fig. 3).

**Table 2:** Differences in demographics and clinical parameters between COVID-19 patients with and without Pleural effusion

	PE (***N*** = 153)	Without PE (***N*** = 323)	***P*** value
**characteristics**			
Age, years	62.32 ± 14.32	60.90 ± 13.56	0.296
Gender			
Male	87 (56.86%)	166 (51.39%)	
Female	66 (43.14%)	157 (48.61%)	0.264
Comorbidity			
Diabetes	26 (16.99%)	44 (13.62%)	0.332
Hypertension	49 (32.03%)	92 (28.48%)	0.429
Cardiovascular disease	15 (9.80%)	34 (10.53%)	0.809
Chronic obstructive pulmonary disease	5 (32.68%)	18 (5.57%)	0.280
Cancer	8 (5.22%)	7 (2.17%)	0.132
Chronic renal disease	9 (5.88%)	5 (1.55%)	0.020
Others	18 (11.76%)	80 (24.77%)	0.001
**Signs and symptoms**			
Fever	117 (76.47%)	277 (85.76%)	0.012
Conjunctival congestion	3 (1.96%)	4 (1.24%)	0.686
Nasal congestion	2 (1.31%)	2 (0.62%)	0.597
Headache	6 (3.92%)	14 (4.33%)	0.834
Cough	99 (64.71%)	208 (64.40%)	< 0.0001
Sputum	52 (33.99%)	93 (28.29%)	0.250
Sore throat	7 (4.58%)	23 (7.12%)	0.286
Fatigue	58 (37.91%)	131 (40.56%)	0.581
Hemoptysis	2 (1.31%)	5 (1.55%)	0.837
Short breath	65 (42.48%)	100 (30.96%)	0.014
Nausea/vomiting	12 (7.84%)	25 (7.74%)	0.969
Musculoarthralgia	10 (6.54%)	22 (6.81%)	0.911
Respiratory rate > 24 breaths per min	24 (15.69%)	35 (10.84%)	0.134
Heart rate ≥ 125 beats per min	3 (1.96%)	147 (45.51%)	< 0.0001
Systolic blood pressure < 90 mmHg	4 (2.61%)	10 (3.10%)	1.000
**Laboratory findings**			
White blood cell count, × 10^9^ per L	6.94 ± 3.31	6.26 ± 2.99	0.026
Neutrophils count, × 10^9^ per L	5.35 ± 3.19	4.59 ± 3.01	0.012
Lymphocyte count, × 10^9^ per L	1.02 ± 0.74	1.16 ± 0.70	0.043
Platelet count,, × 10^9^ per L	209.33 ± 85.92	239.74 ± 99.70	0.001
C-reactive protein, mg/L	48.80 (15.94–89.95)	21.75 (6.63–58.13)	< 0.001
Hemoglobin, g/L	118.73 ± 19.64	124.72 ± 29.27	0.022
Prothrombin time, s	12.10 (11.45–12.65)	12.00 (11.50–12.50)	0.401
Alanine aminotransferase, U/L	29 (18–49)	27 (18–46)	0.714
Aspartate transaminase, U/L	32 (21–44)	28 (20–40)	0.096
Total bilirubin, μmol/L	12.22 ± 6.46	11.87 ± 6.27	0.580
Urea, mmol/L	6.88 ± 6.72	6.92 ± 19.13	0.980
Creatine kinase, U/L	102.50 ± 122.85	98.55 ± 182.87	0.809
Creatine, μmol/L	61 (51–76)	60 (50–70)	0.237
Lactic dehydrogenase, U/L	312 (225–409)	265 (215–333)	0.001
eGFR, mL/min	97.03 (84.82–106.31)	98.00 (88.20–108.01)	0.305
D-dimer, mg/L	1.78 (0.71–5.78)	0.87 (0.50–2.28)	< 0.0001
Partial pressure of oxygen, mmHg	84.09 ± 39.19	99.81 ± 45.46	0.001
Oxygen saturation, %	95 (92–98)	97 (95–99)	0.001
**Timeline after onset of illness**			
Time from illness onset to hospital admission, days	10.88 ± 5.45	9.89 ± 5.63	0.072
Length of hospital stay, days	27.76 ± 5.45	18.91 ± 0.97	< 0.0001
**Timeline after onset of illness**			
Time from illness onset to hospital admission, days	10.88 ± 5.45	9.89 ± 5.63	0.072
Length of hospital stay, days	27.76 ± 5.45	18.91 ± 0.97	< 0.0001
**Disease type**			
Common	0.00 (0.00%)	18 (5.57%)	
Severe	87 (56.86%)	276 (85.45%)	
Critical	66 (43.14)	29 (8.98%)	< 0.0001
**Survival**			
Alive	143 (93.46%)	320 (99.07%)	
Dead	10 (5.54%)	3 (0.93%)	0.001

### Risk factors for critical COVID-19

**Fig. 4:**
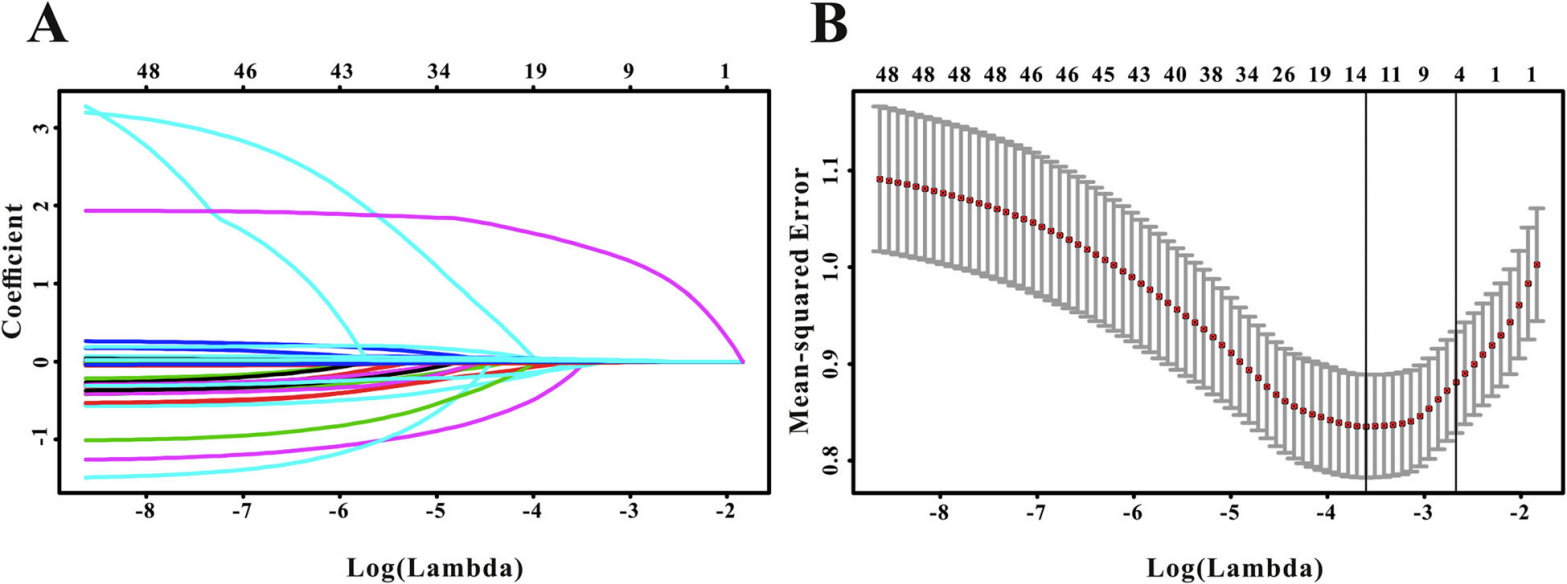
Risk factors were identified by LASSO regression to predict critical type in patients with COVID-19. **a**: LASSO coefficient profiles of the non-zero parameters of COVID-19. **b**: Mean-Squared Error curve of the lowest point in the red line corresponds to a four-variable model

**Fig. 5:**
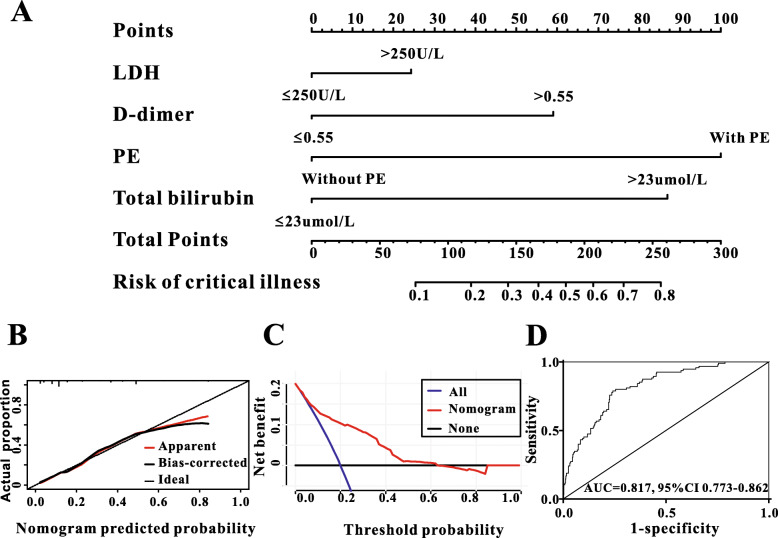
The discriminative power and calibration of the nomogram for predicting critical type in COVID-19 patients. **a**: A nomogram, containing LDH, D-dimer, PE and TBIL was created to predict critical type of COVID-19. **b**: The predicted probabilities by nomogram were coincident with the actual outcomes, indicating the calibration of the nomogram was good. **c**: decision curve analysis (DCA) highlighted the clinical utility of the nomogram. **d**: ROC curve exhibited good discriminative power (AUC = 0.817) for predicting critical type of COVID-19

A total of 48 variables were subjected to the LASSO regression analysis, and the results showed that PE, LDH, D-dimer and TBIL were significantly related to incidence of critical COVID-19 when the partial likelihood deviance was smallest (Fig. 4a and b). A model containing LDH, D-dimer, PE and TBIL, in the form of nomogram, was created to predict the progression to critical condition in COVID-19 patients (Fig. 5a). The risk predicted by the nomogram was virtually consistent with the actual outcomes, indicating the nomogram was well-calibrated (Fig. 5b). Then, decision curve analysis (DCA) was drawn to assess the clinical utility of the nomogram, and if it is clinically useful for the identification of patients who would progress to critical condition (Fig. 5c). Finally, ROC analysis was performed to evaluate the discriminative power of the nomogram. The nomogram exhibited good discriminative performance at AUC of 0.817 for predicting the progression to critical COVID-19 (Fig. 5d).

## Discussion

COVID-19 was diagnosed based on the patients’ contact history, clinical presentations, imaging findings and laboratory results [7, 14, 16]. Chest CT plays an important role in the initial diagnosis of COVID-19. Typical chest CT findings in patients with COVID-19 principally included multiple bilateral patchy ground-glass opacities in lobules with peripheral distribution [7]. Pleural effusion is very commom with the pathological accumulation of fluid in the pleural space. There are many causes of pleural effusion, including viral pleuritis, congestive heart failure or cancer [17]. Patients with a non-malignant pleural effusion have a one-year mortality in the range of 25 to 57% [18]. A recent study found that pleural effusion occurred in 10.3% COVID-19 patients and those refractory patients had a higher incidence of pleural effusion than general COVID-19 patients, suggesting a more obviously inflammatory response in the lung [19]. However, no clinical studies with larger sample size have especially focused on COVID-19 complicated with PE and the implication of PE is underestimated in clinical practice. To our knowledge, this was the first clinical study to examine the imaging features and clinical characteristics of COVID-19 with PE. Our study demonstrated that PE was an uncommon imaging sign and its presence signified unfavorable clinical outcomes.

Typical CT findings of COVID-19 included peripherally distributed multifocal ground-glass opacities plus patchy consolidations, with a potential to involve posterior parts or lower lobes [7]. In the present study, 261 subjects with COVID-19 had PE, including outpatients and inpatients. The incidence rate was 7.09% (261/3679), which was different from that of Severe Acute Respiratory Syndrome. Previous study revealed that Severe Acute Respiratory Syndrome patients radiologically presented more frequently with “ground-glass” changes without PE [20]. Meanwhile, CT findings in 153 inpatients with PE showed that PE was bilateral in most patients (65.36%). COVID-19 patients with PE presented with different types of pulmonary lesions: ground glass opacities, pulmonary consolidation, and liner opacities. Some patients were complicated with pleural thickening (20.26%), pericardial effusion (7.84%) and pulmonary emphysema (5.23%). All aforementioned imaging findings suggested that COVID-19 patients with PE had more involved pulmonary changes.

Common symptoms of COVID-19 included fever, cough, myalgia and fatigue [16]. Compared to patients without PE, COVID-19 patients with PE exhibited more specific symptoms, such as high fever, worse cough and breath shortness. Previous researches demonstrated that evidently decreased lymphocytes, increased platelets, CRP, LDH and D-dimer in COVID-19 patients might indicate that inflammation was severe and disease might deteriorate [2, 21–23]. Our study showed that changes of these indicators were more conspicuous in PE group than in none-PE group. Meanwhile, the partial pressure of oxygen and oxygen saturation were significantly lower in PE group than in none-PE group. PE might substantially inhibit the respiratory function and lower the partial pressure of oxygen and oxygen saturation, eventually exacerbating acute respiratory distress syndrome in patients with severe or critical COVID-19.

Severe COVID-19 patients tended to rapidly progress to acute respiratory failure, acute respiratory distress syndrome, metabolic acidosis, coagulopathy, and septic shock. Early identification of risk factors for severe COVID-19 could lead to prompt supportive care and early admission to the intensive care unit [24]. Hasley et al [25] reported that the presence of bilateral PE was an independent predictor for short-term mortality in patients with community-acquired pneumonia. A study examined patients with MERS-CoV and found that the presence of PE and higher chest radiographic scores were indicative of poor prognosis and higher short-term mortality [26]. In our series, no COVID-19 patients with PE were of general type. Moreover, the median length of hospital stay was longer in PE group than in none-PE group (*P* < 0.0001). The mortality rate was significantly higher in patients with PE than in their counterparts without PE (5.54% & 0.93%, *P* = 0.001). COVID-19 patients with PE might had poor prognosis, suggesting a more obviously inflammatory response in the lung. Early diagnosis and timely and proper treatment for those patients might have satisfactory effect.

Meanwhile, the independent risk factors for critical illness were also screened. The results of the LASSO regression analysis showed that LDH, D-dimer, PE and TBIL were significant risk factors associated with critical COVID-19. Then, we used a model containing LDH, D-dimer, PE and TBIL, in the form of nomogram to predict the progression to critical condition in COVID-19 patients. The risk predicted by the nomogram was virtually consistent with the actual outcomes, indicating combination of the four indicators promised to work better in the prediction of progression to critical condition. In line with our findings, the results by Mo PZ et al. [17] showed that 85 refractory patients had higher levels of maximum temperature among fever cases, higher incidence of breath shortness and anorexia, severer disease assessment on admission, high levels of neutrophils, aspartate aminotransferase (AST), LDH and CRP, lower levels of platelets and albumin, and higher incidences of bilateral pneumonia and PE (45.2%). Also, the Chinese COVID-19 diagnosis and treatment plan (trial version 8) recommended dropping lymphocytes, rising inflammatory factors (e.g., IL-6, CRP), increasing LDH and rapidly progressive pulmonary changes are the predictive factors for severe and critical COVID-19. Now, we used predictive model based on PE and other clinical features to identify COVID-19 patients with critical condition and analyzed PE were significantly related to incidence of critical COVID-19. Therefore, we suggested strongly that PE might be included as a complementary risk factor for the identification of severe and critical COVID-19.

This study had some limitations. First, the study was a single-setting study without external validation cohort. Second, due to a small amount of effusion, thoracentesis could not be performed in COVID-19 patients with PE. Unfortunately, the laboratory findings of pleural effusion were not available. Biochemical analyses of PE caused by COVID-19 needed further study. Third, some patients might have self-medication before hospital admission, which could affect the results of CT images. However, this study focused on the clinical features of COVID-19 complicated with pleural effusion and we hope that our results could help clinicians better evaluate and manage COVID-19 patients with PE.

## Conclusions

Although pleural effusion was uncommon in patients with COVID-19, patients with pleural effusion might have severe inflammation and a poor prognosis. We proposed that pleural effusion should be used as a potential predictor for the progression to severe or critical condition in COVID-19 patients.

## Data Availability

The data generated during the present study are available from the corresponding author on reasonable request.

## Abbreviations

COVID-19: Coronavirus Disease 2019
PE: Pleural effusion
CT: Computed tomography
LDH: Lactic dehydrogenase
TBIL: Total bilirubin
SARS-cov-2: Severe adult respiratory syndrome coronavirus
IQR: Interquartile range
AUC: Area under curve
CRP: C-reactive protein
PO_2_: Partial pressure of oxygen
SpO_2_: Oxygen satueation
